# Pretreatment of microcrystalline cellulose in organic electrolyte solutions for enzymatic hydrolysis

**DOI:** 10.1186/1754-6834-4-53

**Published:** 2011-11-19

**Authors:** Xiao-fei Tian, Zhen Fang, Dan Jiang, Xi-yan Sun

**Affiliations:** 1 Chinese Academy of Sciences, Biomass Group, Laboratory of Tropical Plant Resource Science, Xishuangbanna Tropical Botanical Garden, 88 Xuefulu, Kunming, Yunnan Province, 650223, China; 2 Graduate University of Chinese Academy of Sciences, 19(A) Yuquan Road, Beijing, 100049, China; 3 University of Science, and Technology of China, 96 Jinzhai Road, Hefei, Anhui Province, 230026, China

## Abstract

**Background:**

Previous studies have shown that the crystalline structure of cellulose is negatively correlated with enzymatic digestibility, therefore, pretreatment is required to break down the highly ordered crystalline structure in cellulose, and to increase the porosity of its surface. In the present study, an organic electrolyte solution (OES) composed of an ionic liquid (1-allyl-3-methylimidazolium chloride ([AMIM]Cl)) and an organic solvent (dimethyl sulfoxide; DMSO) was prepared, and used to pretreat microcrystalline cellulose for subsequent enzymatic hydrolysis; to our knowledge, this is the first time that this method has been used.

**Results:**

Microcrystalline cellulose (5 wt%) rapidly dispersed and then completely dissolved in an OES with a molar fraction of [AMIM]Cl per OES (χ _[AMIM]Cl_) of greater than or equal to 0.2 at 110°C within 10 minutes. The cellulose was regenerated from the OES by precipitation with hot water, and enzymatically hydrolyzed. As the χ _[AMIM]Cl _of the OES increased from 0.1 to 0.9, both the hydrolysis yield and initial hydrolysis rate of the regenerated cellulose also increased gradually. After treatment using OES with χ _[AMIM]Cl _of 0.7, the glucose yield (54.1%) was 7.2 times that of untreated cellulose. This promotion of hydrolysis yield was mainly due to the decrease in the degree of crystallinity (that is, the crystallinity index of cellulose I).

**Conclusions:**

An OES of [AMIM]Cl and DMSO with χ _[AMIM]Cl _of 0.7 was chosen for cellulose pretreatment because it dissolved cellulose rapidly to achieve a high glucose yield (54.1%), which was only slightly lower than the value (59.6%) obtained using pure [AMIM]Cl. OES pretreatment is a cost-effective and environmentally friendly technique for hydrolysis, because it 1) uses the less expensive OES instead of pure ionic liquids, 2) shortens dissolution time, 3) requires lower energy for stirring and transporting, and 4) is recyclable.

## Background

Renewable lignocellulosic biomass is the most abundant organic material on the earth, and has been widely used as sustainable raw material for the production of biofuels and platform chemicals [1]. Enzymatic saccharification is considered as one of the most promising ways to break down lignocellulosic material into sugars for fermentation and chemical conversion [2]. Previous studies have shown that the degree of crystallinity of lignocellulosic biomass, which is related to the crystalline structure of the cellulose component, is negatively correlated with enzymatic digestibility [3]. Therefore, pretreatment, serving as the first step in the saccharification of biomass, is required to break down the highly ordered crystalline structure of cellulose [4], and to increase the porosity of its surface [5].

A number of conventional approaches have been widely used for pretreatment, including physical (for example, grinding, ball-milling, and irradiation), chemical (involving use of, for example, alkalis, dilute acids, oxidizing agents, and organic solvents), physicochemical (for example, steam explosion, ammonia-fiber explosion, hydrothermolysis, and wet oxidation), and biological pretreatment methods, and combinations of these [5]. Recently, ionic liquids (ILs) have been successfully used to dissolve cellulose at room temperature, to form IL + cellulose solutions [6-8]. The structure of cellulose regenerated from these solutions by precipitation was essentially amorphous and porous, which made the subsequent enzymatic hydrolysis more efficient [8-11]. ILs are non-volatile with a low vapor pressure, and can be easily separated by distillation or condensation [12]. Pretreatment with ILs is considered an environmentally friendly alternative to conventional pretreatment methods [13]. Although pretreatment with ILs is a viable method, it faces three major challenges [14,15]: 1) the slow rate of dissolution within these liquids [16] means that it takes a long time for complete dissolution of the biomass; 2) the high viscosity of the solutions [17] causes agglomeration of cellulose and a resulting high consumption of energy for stirring; and 3) the high cost of ILs [15] makes them uneconomic for commercial use.

To overcome these drawbacks, Sui *et al. *prepared a homogeneous cellulose solution by adding N,N-dimethylformamide (DMF) into a 1-allyl-3-methylimidazolium chloride;([AMIM]Cl). Additional DMF component reduced the viscosity of the whole solvent at room temperature [18]. Luo *et al. *reported that mixtures composed of dipolar aprotic intercrystalline swelling agents (for example, acetone, dioxane, pyridine, *N*-oxide, *N*-methyl pyridine, and hexamethylphosphoramide), and ILs can also dissolve wood pulp [19]. Rinaldi developed a series of solvent systems called 'organic electrolyte solutions' (OESs), which contained a polar aprotic solvent (for example, DMF, *N,N*-dimethylacetamide, 1, 3-dimethyl-2-imidazolidinone, and dimethyl sulfoxide (DMSO)) and only a small molar fraction of ILs; these solutions had a strong ability to dissolve cellulose quickly [15]. Because of their potential novel properties, OESs might be useful, environmentally friendly, and cost-saving solvents for pretreatment. However, no study has yet been performed to characterize and hydrolyze OES-pretreated cellulose.

The objective of this study was to evaluate the pretreatment effectiveness of dissolution and subsequent regeneration of cellulose in an OES for enzymatic hydrolysis. Therefore, we set out to: 1) design a simple OES system using ([AMIM]Cl and DMSO) for cellulose dissolution; 2) monitor the physical changes in the regenerated cellulose that was precipitated by hot water from the cellulose + OES mixture at different molar fractions of [AMIM]Cl/OES (χ _[AMIM]Cl_), and 3) determine the hydrolysis rate and the yield of the cellulose after regeneration.

## Results and discussion

In total, 22 cellulose samples pretreated with OES at various molar fractions (χ _[AMIM]Cl _from 0 to 1.0) were enzymatically hydrolyzed (396 runs) for different times (from 3 to 72 hours). Enzymatic hydrolysis of the untreated cellulose (36 runs), and cellulose pretreated only with hot water (36 runs) was also conducted for comparison.

### Viscosity of organic electrolyte solutions

The parameters of the Vogel-Fulcher-Tammann (VFT) and Arrhenius models were estimated by fitting the reported viscosity data [20,21] (Table 1). Based on these models, the calculated viscosities of pure [AMIM]Cl (χ _[AMIM]Cl _= 1.0) and pure DMSO (χ _[AMIM]Cl _= 0) were 16.35 and 0.61 cP at 110°C, respectively. Based on the Grunberg-Nissan mixing law, the viscosity of the OES preparations with χ _[AMIM]Cl _from 0.1 to 0.9 (in steps of 0.1) was calculated as 0.85, 1.18, 1.64, 2.27, 3.16, 4.39, 6.10, 8.47, and 11.77 cP, respectively, at the dissolution temperature of 110°C. Compared with pure [AMIM]Cl, the OES had a lower viscosity because of the additional DMSO component. This could be an advantage as it may avoid agglomeration during rapid dispersing of cellulose in the OES. Moreover, a practical flow-process system can be easily used with the low-viscosity OES to pretreat cellulose, which could save energy in pipeline transport and stirring the samples.

**Table 1:** Estimated parameters for Vogel-Fulcher-Tammann (VFT) and Arrhenius models (equations 4 and 5).

Model	**χ**^**2**^	***R***^**2**^	Parameter	Value	Error
VFT	7.95	0.99	*K*	0.17	0.073
			*Θ*	56.27	5.86
			*B*	762.53	86.79
Arrhenius	0.0005	0.99	*A*	0.010	0.012
			*E*	-35.88	379.97
			*R*	-0.023	0.23

### Cellulose dissolution and regeneration

**Figure 1:**
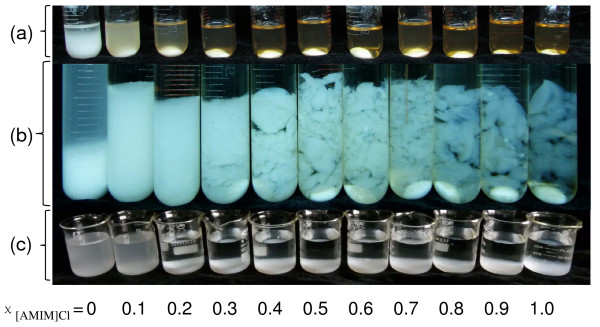
**Images of dissolved and regenerated cellulose**. (a) Dissolved cellulose in organic electrolyte solution (OES), (b) cellulose regenerated by hot water, and (c) precipitation of the regenerated cellulose after washing five times with water. The molar fraction of 1-allyl-3-methylimidazolium chloride ([AMIM]Cl)/OES (**χ **_[AMIM]Cl_) is from 0 to 1.0. (a, b) Magnetic bars are present at the bottom of the tubes.

The cellulose solutions and OES were prepared by stirring the mixturs of crystalline cellulose and OES at 110°C (detailed pretreatment conditions are summarized in Table 2). The cellulose dispersed instantaneously, and rapidly dissolved within 10 minutes in OES with χ _[AMIM]Cl _of 0.2 to 0.9. However, large amounts of cellulose were suspended at low χ _[AMIM]Cl _values (that is, 0 and 0.1), and agglomerated at the high value (χ _[AMIM]Cl _of 1.0) because of the high viscosity of pure [AMIM]Cl. To ensure all the cellulose was completely dissolved, all mixtures were stirred for another 50 minutes. With pure DMSO (χ _[AMIM]Cl _= 0), there was still precipitation of crystalline cellulose at the bottom of the tube after 60 minutes of mixing (Figure 1a), indicating that the cellulose was insoluble in pure DMSO. When the χ _[AMIM]Cl _was increased to 0.1, the precipitated sample disappeared, but the mixture was totally opaque, owing to the formation of cellulose colloidal granules. As χ _[AMIM]Cl _increased further, the mixtures gradually cleared, and at χ _[AMIM]Cl _≥ 0.3, the mixtures were totally transparent because the cellulose had completely dissolved in the OES.

**Table 2:** Composition of the organic electrolyte solution (OES) and weight ratio of cellulose per ionic liquid (IL) ^a^.

**χ **_**[AMIM]Cl**_^**c**^	Weight, g			Weight ratio of cellulose/IL, g/g
		
	IL	DMSO	**Cellulose**^**b**^	
0	0	3	0.15	N/A
0.1	0.553	2.447	0.15	0.271
0.2	1.01	1.99	0.15	0.149
0.3	1.396	1.604	0.15	0.107
0.4	1.725	1.275	0.15	0.087
0.5	2.01	0.99	0.15	0.075
0.6	2.258	0.742	0.15	0.066
0.7	2.478	0.523	0.15	0.0605
0.8	2.746	0.254	0.15	0.0546
0.9	2.884	0.156	0.15	0.052
1.0	3	0	0.15	0.05

^a^Weight ratio of cellulose/IL = (gram of cellulose added in the OES)/(gram of 1-allyl-3-methylimidazolium chloride ([AMIM]Cl)/in the OES).

^b^Weight percentage of (cellulose/(IL + dimethylsulfoxide)) = 5%.

^c^Molar fraction of 1-allyl-3-methylimidazolium chloride ([AMIM]Cl)/OES.

**Figure 2:**
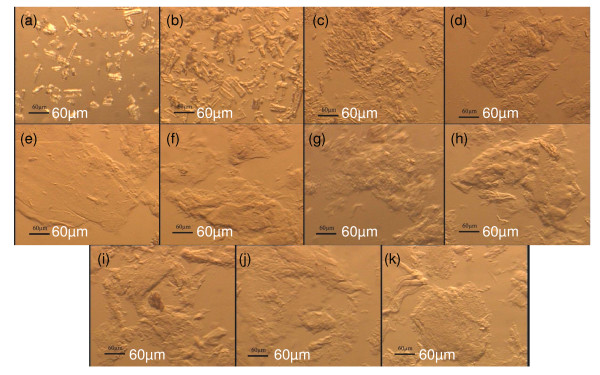
**Micrographs of cellulose after regeneration from the organic electrolyte solution (OES)**. (a) Untreated cellulose, and (b-k) cellulose pretreated with OES at different values of **χ **_[AMIM]Cl _(molar fraction of 1-allyl-3-methylimidazolium chloride ([AMIM]Cl)/OES): (b) 0.1, (c) 0.2, (d) 0.3, (e) 0.4, (f) 0.5, (g) 0.6, (h) 0.7, (i) 0.8, (j) 0.9, (k) 1.0.

It has been reported previously [13,22] that cellulose solubility in pure [AMIM]Cl was less than 0.18 (w/v) or 0.17 (w/w). In the present study, we found that when χ _[AMIM]Cl _was equal to 0.1, 0.2, and 0.3, the weight ratio of (cellulose/[AMIM]Cl) in the OES was approximately 0.27, 0.15, and 0.11 (w/w), respectively (Table 2). When the weight ratio of (cellulose/[AMIM]Cl) in the OES was equal to 0.27 (> 0.17), the cellulose did not dissolve, but rather swelled (Figure 2b). Therefore, there was no evidence that DMSO promoted cellulose dissolution in the OES.

Zhang *et al. *proposed a dissolution mechanism of cellulose in pure [AMIM]Cl, suggesting that the free Cl^- ^anions associate with cellulose hydroxyl protons and the free cations complex with the cellulose hydroxyl oxygens, leading to the disruption of hydrogen bonding in the cellulose and its consequent dissolution [23]. DMSO is unable to donate cations and anions, so it has no positive effect in promoting cellulose dissolution. Therefore, in the OES dissolution system, a lower concentration of [AMIM]Cl with fewer Cl^- ^anions and cations had a reduced ability to destroy the cellulose crystal structure for dissolution.

The dissolution ability of cellulose in the OES can also be interpreted by the hydrogen bond-accepting ability (basicity) of the system, as measured by the Kamlet-Taft parameter β [15,24]. A system with a higher β value represents a higher hydrogen bond-accepting ability, which can significantly reduce the crystallinity of cellulose for dissolution [25]. According to this theory, because [AMIM]Cl has a higher β parameter than DMSO, as the DMSO fraction decreased (that is, the χ _[AMIM]Cl _increased from 0 to 1.0), the solubility of cellulose in the OES increased as a result of the increase in the hydrogen bond-accepting ability in the system.

After regeneration using water (an anti-solvent), the cellulose became swollen at χ _[AMIM]Cl _= 0.1, whereas no cellulose gel was formed until the χ _[AMIM]Cl _reached 0.3 (Figure 1b). When χ _[AMIM]Cl _was increased from 0.3 to 1.0, the gel became gradually more agglomerated and transparent. The regenerated cellulose was washed five times with hot water to remove the OES before hydrolysis. After washing (Figure 1c), the crystalline cellulose particles still remained and were suspended at χ _[AMIM]Cl _of 0 and 0.1, but they became gel-formed cellulose blocks and precipitated out of solution at χ _[AMIM]Cl _of 0.2 to 1.0. At χ _[AMIM]Cl _of greater than or equal to 0.2, the cellulose solubility in the OES increased as a result of the reduction in the parameter β (interference of [AMIM]Cl by DMSO). This phenomenon can also be seen in the micrographs of the regenerated cellulose (Figure 2a-k), in which the regenerated cellulose is seen to be amorphous at χ _[AMIM]Cl _of greater than or equal to 0.2.

**Figure 3:**
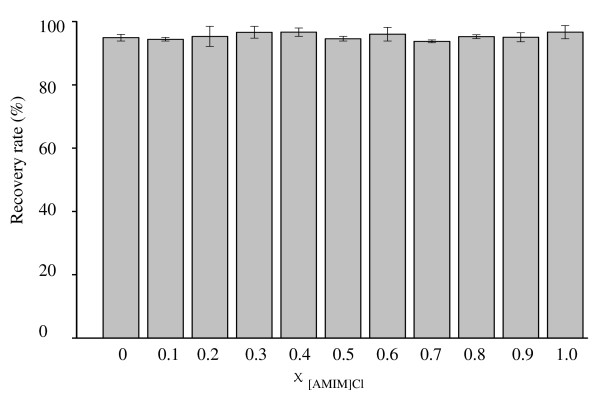
**Recovery rate of the regenerated cellulose pretreated with organic electrolyte solution (OES) at different values of χ _[AMIM]Cl _(molar fraction of 1-allyl-3-methylimidazolium chloride ([AMIM]Cl)/OES)**.

The recovery rate of cellulose regenerated from the OES at different values of χ _[AMIM]Cl _was assessed (Figure 3), and clearly indicated there was no significant difference between the recovery rates (*P *= 0.3229) at χ _[AMIM]Cl _from 0 to 1.0. The average recovery rate was 95.37 ± 1.41%. Additionally, χ _[AMIM]Cl _had a slight but insignificant effect on the recovery rate (*r *= 0.14, *P *= 0.68). This loss of cellulose was mainly due to some tiny cellulose particles being suspended in water during washing, which were difficult to recover.

### Degree of crystallinity

**Figure 4:**
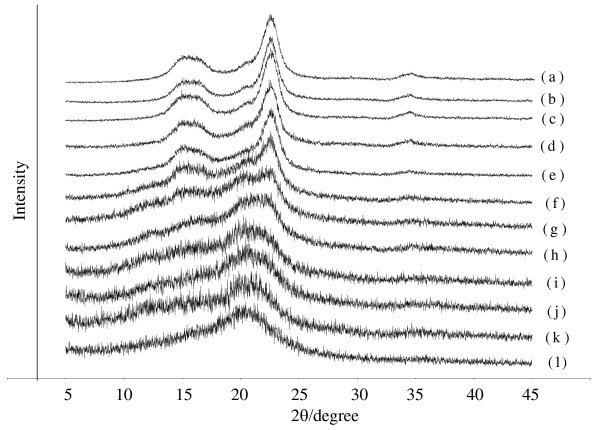
**X-ray diffraction (XRD) patterns of cellulose with and without pretreatment**. (a) Untreated microcrystalline cellulose, and (b-k) cellulose pretreated with organic electrolyte solution (OES) at different values of χ _[AMIM]Cl _(molar fraction of 1-allyl-3-methylimidazolium chloride ([AMIM]Cl)/OES): (b) 0, (c) 0.1, (d) 0.2, (e) 0.3, (f) 0.4, (g) 0.5, (h) 0.6, (i) 0.7, (j) 0.8, (k) 0.9, (l) 1.0.

The crystal structures of the cellulose had a strong inﬂuence on its hydrolysis kinetics [26]. The crystallinity of the cellulose regenerated from the OES was studied by X-ray diffraction (XRD) analysis. The XRD patterns of untreated microcrystalline cellulose and celluloses regenerated from the OES at χ _[AMIM]Cl _from 0 to 1.0 showed distinct peaks with diffraction angles (2θ) at around 22.6, 20.3, 16.3, and 14.9 degrees for the untreated cellulose (Figure 4), indicating that its crystal structure consisted of celluloses I and II [26]. After dissolution in the OES and subsequent precipitation with ho water, the peak intensities at around 22.6, 16.3, and 14.9 degrees began to decrease gradually, due to the reduction in crystalline cellulose I in the precipitated cellulose. At χ _[AMIM]Cl _of 0.5, the XRD pattern began to flatten because of the presence of the more amorphous cellulose I. As χ _[AMIM]Cl _increased from 0 to 1, the crystallinity index (CI; represents the percentage of crystalline components in the cellulose sample) of cellulose I decreased from 0.834 to -0.319, whereas the CI of cellulose II changed little (Table 3), indicating that the crystalline structure of cellulose I was gradually destroyed as the χ _[AMIM]Cl _rose.

**Table 3:** Effect of χ _[AMIM]Cl_^a ^on the structure features of untreated cellulose, and pretreated cellulose regenerated from organic electrolyte solution (OES).

**χ **_**[AMIM]Cl**_	Crystallinity		Speciﬁc surface area, m^2^/g	^**b**^**DP**_**n**_
			
	CI cellulose I	CI cellulose II		
Untreated	0.80	0.39	2.28	125 ± 1
0	0.83	0.24	3.38	135 ± 26
0.1	0.83	0.29	4.43	130 ± 15
0.2	0.73	0.23	4.43	137 ± 22
0.3	0.67	0.31	3.49	141 ± 24
0.4	0.47	0.26	3.68	148 ± 15
0.5	0.25	0.23	4.80	133 ± 7
0.6	0.22	0.29	4.18	126 ± 0
0.7	0.008	0.28	3.99	146 ± 19
0.8	-0.03	0.36	3.75	127 ± 13
0.9	-0.14	0.33	4.34	155 ± 0
1.0	-0.32	0.26	4.65	129 ± 19

^a^Molar fraction of 1-allyl-3-methylimidazolium chloride ([AMIM]Cl)/OES.

^b^Data were based on two independent experiments.

**Figure 5:**
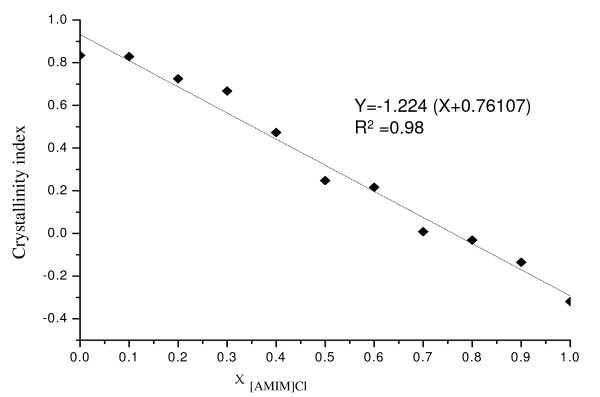
**Correlation of crystallinity index of regenerated cellulose pretreated with organic electrolyte solution (OES) at different values of χ _[AMIM]Cl _(molar fraction of 1-allyl-3-methylimidazolium chloride ([AMIM]Cl)/OES)**.

It has been reported that cellulose I is much more resistant to hydrolyzation than cellulose II or amorphous cellulose [27,28], therefore the enhancement in hydrolysis rate and yield of regenerated cellulose in the following hydrolysis procedure was due to the presence of more amorphous cellulose I in the restructured cellulose after pretreatment. There was a strong negative linear correlation between the CI of the regenerated cellulose and the χ _[AMIM]Cl_, with a correlation coefficient of 0.98 (Figure 5). Previous studies have developed several methods to prepare certain crystal forms of cellulose with different degrees of crystallinity. For example, the cellulose structure can be reformed by simply pouring cellulose + IL solution into a precipitation agent, or by adding the precipitation agent into the cellulose + IL solution [29,30]. In this study, we have identified another efficient method to prepare cellulose with controlled CI, using dissolution in OES and precipitation with hot water.

### Surface area and degree of polymerization

Besides crystallinity, the enzymatic hydrolysis of lignocellulose is influenced by other factors, such as available surface area, degree of polymerization (DP), moisture content, and lignin content [31]. To examine the effects of surface area and DP on enzymatic hydrolysis, the Bruner, Emmett, and Teller (BET) method and the photocolorimetric method developed by Zhang and Lynd [32] were used to determine the speciﬁc surface area and the number-average DP (DP_n_), respectively. The speciﬁc surface area of the cellulose regenerated from the OES at different χ _[AMIM]Cl _values from 0 to 1.0 (with steps of 0.1), was, respectively, 1.48, 1.94, 1.94, 1.53, 2.76, 2.10, 1.83, 1.75, 1.64, 1.90, and 2.04 times that of the untreated cellulose (Table 3), indicating that the pretreatment could enhance the specific surface area of cellulose. Furthermore, no close relationship was found between χ _[AMIM]Cl _and speciﬁc surface area (*r *= 0.49, *P *= 0.13).

Compared with untreated cellulose, the DP of the regenerated cellulose had a slight but insignificant increase (*P = *0.65), which indicated that cellulose pretreated with OES resulted in little degradation. It has been reported that some pretreatment methods such as acid hydrolysis (even with dilute acid) resulted in significant decomposition of polysaccharides, which led to lower recovery of pretreated biomass [12]. Using the OES pretreatment, there was little cellulose decomposition, and hence a higher recovery rate of regenerated cellulose (95.37 ± 1.41%).

### Enzymatic hydrolysis of regenerated cellulose

**Figure 6:**
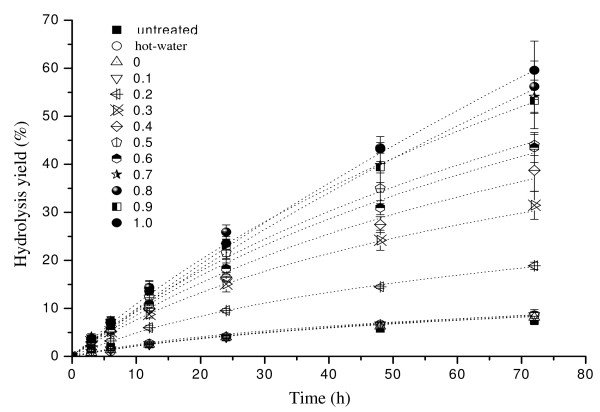
**Hydrolysis yield in 0, 3, 6, 12, 24, 48, and 72 hours for untreated cellulose, and cellulose pretreated with hot water and organic electrolyte solution at different values of χ _[AMIM]Cl _(molar fraction of 1-allyl-3-methylimidazolium chloride ([AMIM]Cl)/OES)**.

Because hot water has been used previously as a pretreatment method [33], we needed to investigate whether the hot-water rinsing of the regenerated cellulose contributed to the enhancement of enzymatic hydrolysis rather than OES dissolving. Hydrolysis tests (468 runs) were conducted for 3, 6, 9, 12, 24, 48, and 72 hours for the untreated (36 runs), hot-water-treated (36 runs), and regenerated (pretreated with the OES; 396 runs) cellulose samples (Figure 6, Table 4). We then compared the rate retardation constant (*k*) and initial hydrolysis rate (*v*_0_) between untreated and hot-water-treated cellulose, and found that, similar to the previous work [33], hot-water rinsing of regenerated cellulose enhanced enzymatic hydrolysis (*P *< 0.05). To control for the influence of hot-water rinsing, its hydrolysis data were used as a blank reference for the comparison with those of the OES-pretreated cellulose (Table 4).

**Table 4:** Effect of χ _[AMIM]Cl_^Ia ^on the enzymatic hydrolysis yield of pretreated cellulose by organic electrolyte solution (OES).

Time, hours	**Hydrolysis yield (%) at different values of χ **_**[AMIM]C**_^**lb**^												
	
	Untreated cellulose	Hot-water	0	0.1	0.2	0.3	0.4	0.5	0.6	0.7	0.8	0.9	1.0
3	1.28 ± 0.17^d, e^	0.78 ± 0.07^e^	0.85 ± 0.075^e^	0.91 ± 0.21^e^	2.22 ± 0.28^d^	3.04 ± 0.27^c^	3.02 ± 0.20^c^	3.34 ± 0.38^b, c^	3.17 ± 0.40^c^	4.15 ± 0.56^a^	3.81 ± 0.13^a^	3.63 ± 0.49^a.b^	3.62 ± 0.30^ab^
6	1.88 ± 0.19^cd^	1.03 ± 0.12^e^	1.33 ± 0.04^e^	1.41 ± 0.23^d, e^	3.22 ± 0.47^c^	5.13 ± 0.49^b^	5.24 ± 0.33^b^	6.77 ± 0.42^a^	5.64 ± 0.54^b^	7.40 ± 0.75^a^	7.35 ± 0.29^a^	7.01 ± 1.27^a^	7.06 ± 0.60^a^
12	2.49 ± 0.38^f^	2.68 ± 0.11^f^	2.5 ± 0.12^f^	2.51 ± 0.33^f^	5.98 ± 0.49^e^	8.74 ± 0.75^d, e^	10.02 ± 0.55^c, d^	13.88 ± 2.12^b^	10.85 ± 0.62^c^	13.9 ± 1.68^a, b^	14.86 ± 0.7^a^	13.57 ± 2.27^a.b^	13.62 ± 0.84^a.b^
24	3.96 ± 0.30^f^	4.09 ± 0.12^f^	4.02 ± 0.27^f^	4.03 ± 0.38^f^	9.53 ± 0.44^e^	15.05 ± 1.63^d^	16.48 ± 0.97^d^	22.35 ± 3.53^b^	18.29 ± 1.17^c^	23.18 ± 2.78^a, b^	24.79 ± 0.77^a^	23.07 ± 2.85^a.b^	23.53 ± 1.41^a.b^
48	5.76 ± 0.60^g^	6.69 ± 0.39^g^	6.28 ± 0.47^g^	6.62 ± 0.64^g^	14.49 ± 0.37^f^	24.18 ± 2.1^e^	27.43 ± 1.64^e^	36.69 ± 5.03^c^	30.98 ± 1.47^d^	39.81 ± 3.57^b^	44.34 ± 2.43^a^	39.38 ± 4.5^b^	43.19 ± 2.6^a, b^
72	7.50 ± 0.87^f^	8.62 ± 0.70^f^	8.12 ± 1.07^f^	8.52 ± 1.22^f^	18.87 ± 0.76^e^	31.45 ± 2.92^d^	38.76 ± 4.37^c^	47.51 ± 5.77^c^	43.5 ± 3.1^c^	54.08 ± 3.46^ab^	53.03 ± 8.92^a, b^	53.23 ± 5.84^b^	59.57 ± 6.07^a^

^la^Molar fraction of 1-allyl-3-methylimidazolium chloride ([AMIM]Cl)/OES.

^lb^In total, 468 runs were conducted. The data in each cell are based on six runs from two parallel experiments, each of which gave three parallel tests, respectively. Mean values with the same superscript letters on the same horizontal row are not significantly different. The 95% confidence level was set at *P *< 0.05.

Similar to results from previous studies [9,10,22,26], the enzymatic hydrolysis yield for the cellulose samples pretreated with pure [AMIM]Cl (χ _[AMIM]Cl _= 1.0) was significantly increased; the highest hydrolysis yield was 59.6% after 72 hours, which was 6.9 times that of the hot-water-treated cellulose and 7.9 times that of the original cellulose. Our results clearly showed that after pretreatment with OES at χ _[AMIM]Cl _from 0.2 to 1.0, the hydrolysis yield improved gradually. Additionally, no significant change was found for hydrolysis yield (54 to 59.6%) at χ _[AMIM]Cl _= 0.7 to 1.0 (*P *= 0.21) after 72 hours, thus indicating that DMSO had little negative effect on hydrolysis yield when χ _[AMIM]Cl _was greater than or equal to 0.7.

**Figure 7:**
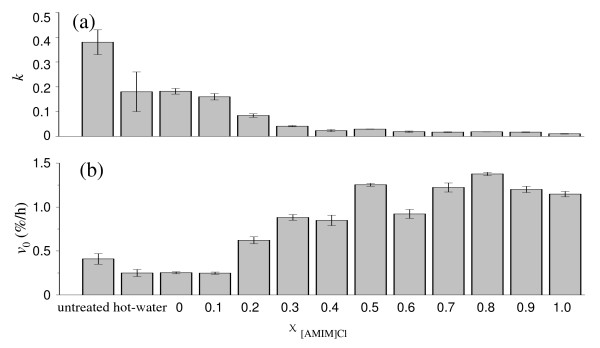
**Influence of at different values of χ _[AMIM]Cl _(molar fraction of 1-allyl-3-methylimidazolium chloride ([AMIM]Cl)/OES) on (a) rate retardation constant (*k*), and (b) initial hydrolysis rate (*v_0_*) for enzymatic hydrolysis of cellulose with and without pretreatment**.

Regression analysis of the experimental hydrolysis yield was performed based on the empirical equation proposed by Ohmine *et al. *[34]. The initial hydrolysis rate (*v*_0_) was enhanced as χ _[AMIM]Cl _increased from 0 to 1.0 (Figure 7b, Table 5). At the same time, the rate retardation constant (*k*) had a tendency to decline (Figure 7a, Table 5). The initial hydrolysis rate of the cellulose pretreated at χ _[AMIM]Cl _from 0 to 1.0 (with steps of 0.1) was, respectively, 1.00, 1.00, 2.48, 3.52, 3.40, 5.00, 3.68, 4.88, 5.48, 4.8, and 4.56 times that of the hot-water-treated cellulose. Meanwhile, the corresponding *k *value was, respectively, 1.00, 0.89, 0.44, 0.22, 0.11, 0.17, 0.06, 0.11, 0.11, 0.11, and 0.06 times. These results showed that after pretreatment with OES, the increase in enzymatic hydrolysis yield was not only due to the increase in initial hydrolysis rate but also to the decline in the rate retardation constant. There were no significant difference in *v_0_, and k *values at χ _[AMIM]Cl _0.7 to 1.0 (Table 5). When χ _[AMIM]Cl _was greater than or equal to 0.7, a further increase in [AMIM]Cl had little effect on hydrolysis yield.

**Table 5:** Kinetics parameters of enzymatic hydrolysis of cellulose pretreated with organic electrolyte solution (OES) at different values of χ _[AMIM]Cl_^Ia ^[34].

**χ **_**[AMIM]Cl**_	***v***_**0**_^**Ib**^	***k***^**Ib**^	***R***^**2**^
Untreated	0.41 ± 0.06^c^	0.38 ± 0.05^a^	0.99
Hot water	0.25 ± 0.04^c^	0.18 ± 0.08^b^	0.99
0	0.25 ± 0.01^c^	0.18 ± 0.01^b^	0.99
0.1	0.25 ± 0.01^c^	0.16 ± 0.01^b^	0.99
0.2	0.62 ± 0.04^c^	0.08 ± 0.01^b, c^	0.99
0.3	0.88 ± 0.03^b^	0.04 ± 0.002^c, d^	0.99
0.4	0.85 ± 0.06^b^	0.02 ± 0.003^e^	0.99
0.5	1.25 ± 0.02^a^	0.03 ± 0.0006^d^	0.99
0.6	0.92 ± 0.05^b^	0.01 ± 0.003^e, f^	0.99
0.7	1.22 ± 0.05^a^	0.02 ± 0.002^e, f^	0.99
0.8	1.37 ± 0.02^a^	0.02 ± 0.0005^e, f^	0.99
0.9	1.20 ± 0.04^a^	0.02 ± 0.001^e, f^	0.99
1.0	1.14 ± 0.03^a^	0.01 ± 0.001^f^	0.99

^Ia^Molar fraction of 1-allyl-3-methylimidazolium chloride ([AMIM]Cl)/OES.

^Ib^The data in each cell are based on six runs from two parallel experiments, each of which gave three parallel tests, respectively. Mean values with the same superscript letters on the same horizontal row are not significantly different. The 95% confidence level was set at *P *< 0.05.

Based on the XRD, BET, and DP results, hydrolysis yield rose gradually as χ _[AMIM]Cl _increased from 0.2 to 1.0, which was due to the decline in crystallinity of the cellulose I component rather than to changes in specific surface area or DP, as these changed little and the change was irregular.

A large surface area assists the cellulase in accessing the cellulose for hydrolysis. However, at low values of χ _[AMIM]Cl _(0 and 0.1), the specific surface area was enhanced (respectively, 1.48 and 1.94 times that of untreated cellulose), but the hydrolysis yield increased little (Table 3 Table 4). This may be because the pretreated cellulose still had a highly crystalline structure, which played a more important role in hydrolysis than surface area.

A higher hydrolysis rate and yield were correlated with lower DP cellulose, which had more reducing ends of cellulose available to provide more sites for the exocellulase to begin cleavage [26]. However, no enhanced performance of hydrolysis was found in this study, because the DP changed little (Table 3).

OES has the ability to dissolve cellulose at χ _[AMIM]Cl _of greater than or equal to 0.2. Compared with conventional ILs, the low viscosity of OES promotes cellulose dispersion, and inhibits its agglomeration at a relative low temperature. Thus, an OES with low viscosity can be used to process cellulose continuously in a flow system for industrial applications. Taking into consideration the cost of the solution and the resulting hydrolysis yield, an OES with χ _[AMIM]Cl _of 0.7 was chosen as the ideal solvent for cellulose pretreatment. The viscosity was only 37% that of pure [AMIM]Cl, and the glucose yield (54.08 ± 3.46%) was 7.2 times that of untreated cellulose after 72 hours of hydrolysis.

### Economic advantages

The economic benefits of OES pretreatment include: 1) reduced costs of IL (for example, 70% [AMIM]Cl), 2) less energy required for stirring and transporting of the mixture, owing to the low viscosity of the OES, 3) higher throughput because of the shortened dissolution time, and 4) environmental benefits from the reduction of gas, water, and solid wastes. For industrial use, the OES needs to be separated from the anti-solvent (water) for recycling, which can be achieved easily using commercial distillation technology at reduced pressure [35]. Additionally, some new technologies, such as nanoﬁltration, reverse osmosis, and pervaporation [36], three-phase system precipitation [37], and supercriticalCO_2 _extraction [35,36] have potential applications in recycling of OES.

## Conclusions

We describe a new method for pretreatment of cellulose, using an OES dissolution system composed of an IL ([AMIM]Cl) and an organic solvent (DMSO) to improve the rate and yield of subsequent enzymatic hydrolysis. This OES has the ability to dissolve 5 wt% microcrystalline cellulose in a very short time (≤ 10 minutes) at 110°C with χ _[AMIM]Cl _of greater than or equal to 0.2. After pretreatment with OES with χ _[AMIM]Cl _of 0.7, the crystallinity (cellulose I) decreased by 99%. The initial hydrolysis rate and glucose yield in 72 hours were, respectively, 3 and 7.2 times that of untreated cellulose. The hydrolysis yield was similar to pretreatment with the pure ILs [AMIM]Cl. The promotion of hydrolysis was due to the sharp decline in crystallinity rather than to changes in the specific surface area or DP of cellulose after pretreatment. Further studies are underway to examine recycling of the OES, and the integration of microwave and ultrasonic irradiation in the OES pretreatment to improve the effectiveness of this process.

## Methods

### Preparation of the organic electrolyte solution

The molar fraction χ _[AMIM]Cl _was defined as:

(1) $$\chi \text{[AMIM]Cl}=\frac{\text{Mole}\;\text{of}\;\text{[AMIM]Cl}}{\text{Mole}\;\text{of}\;\text{[AMIM]Cl}+\text{mole}\;\text{of}\;\text{DMSO}},$$

where χ _[AMIM]Cl _at 0 and 1.0 represent the pure DMSO and pure [AMIM]Cl solvents, respectively. [AMIM]Cl (99% purity; Shanghai Chengjie Chemical Co. Ltd, Shanghai, China) was dried in a blast drier (Yuhua DHG, Gongyi, Henan, China) at 90°C for 72 hours before use, and the OES was prepared by mixing the dried [AMIM]Cl with DMSO (99.5% purity; Shantou Xilong Chemical Co. Ltd, Guangdong, China) at different molar fractions (χ _[AMIM]Cl_) from 0.1 to 0.9 at room temperature (Table 2).

### Cellulose dissolution and regeneration

Microcrystalline cellulose (99% purity, particle size 100 μm, density 0.3 g/mL; BioBasic Inc., Shanghai, China) was dried in an oven at 60°C for 24 hours before use. A suspension of cellulose in OES was prepared by adding 0.15 g (5 wt%) of the microcrystalline cellulose into a 20-mL glass-stoppered test tube containing 3.0 g of OES, with a magnetic bar. The tube was placed in an oil bath with magnetic stirrer at 110°C for 60 minutes and 200 rpm to form the cellulose solution. The regenerated cellulose was precipitated in the tubes by adding 15 mL deionzed water at 90°C, and vigorously shaking the tube for 10 seconds. The precipitated cellulose was transferred into a beaker with 50 mL fresh deionized water at 70°C, and washed thoroughly with five changes of deionized water to remove the residual solvent. The cellulose was freeze-dried for 12 hours (Eyela 1200 freeze dryer; Tokyo Rikakikai Co, Ltd, Tokyo, Japan), then the recovered cellulose was weighed and used for subsequent enzymatic hydrolysis. The recovery rate of cellulose was defined as:

(2) $$\text{Recovery rate of cellulose (\% ) = }\frac{\text{Mass}\;\text{of}\;\text{recovered}\;\text{cellulose(g)}}{0.15g}\times 100$$

The reported data are the mean of three replicates.

### Micrographs of cellulose samples

Micrographs were taken of the untreated and regenerated cellulose samples to evaluate any morphological changes in the cellulosic microstructures after OES pretreatment. Cellulose samples (1‰ w/w) were suspended in deionized water on labeled glass microscope slides, then digital photomicrographs were taken using a stereomicroscope (SMZ 1500; Nikon, Tokyo, Japan).

### Crystallinity measurement

CI was determined by diffracted intensity of Cu radiation (1.54 Å, 40 kV, and 200 mA) using an X-ray diffractometer (TTR III; Rigaku, Tokyo, Japan). Each sample (80 mg) was pressed into a lamellar container 20 mm in diameter, and scanned at 10 degrees/min in a range of 2θ values from 5 to 45 degrees. CI was calculated by the empirical equation:

(3) $$\text{CI = }\frac{I_{Cr}-I_{Am}}{I_{Cr}}$$

where *I_Cr _*is the average intensity of crystalline region at 2θ of 22.56 to 22.65 degrees for cellulose I or 21.66 to 21.75 degrees for cellulose II; and *I_Am _*represents the average intensity of the amorphous region at 2θ of 18.96 to 19.05 degrees for cellulose I, or 15.96 to 16.05 degrees for cellulose II [26].

### Viscosity of organic electrolyte solutions

Based on the viscosity data of pure [AMIM]Cl, and DMSO from [20,21], the viscosity (*η*)for [AMIM]Cl and DMSO versus temperature was studied by fitting the VFT equation (equation 4), and Arrhenius model (equation 5), respectively, using Origin software (version 7.5; OriginLab Co. Ltd, Northampton, MA) [38,39]. The equations were as follows.

(4) $$\text{VFT equation}:\;\eta \;\text{(}t\text{)}=K\;\text{exp}\;\text{[}\frac{b}{t+\Theta }\text{]}$$

where *η*(*t*) is the viscosity of [AMIM]Cl, *t *is temperature (°C); and *K, b*, and *Θ *are the coefficients (Table 1).

(5) $$\text{Arrhenius model}:\;\eta \;\text{(}T\text{)}=A\;\text{exp (}\frac{E}{RT}\text{) }$$

where *η*(*T*) is the viscosity for DMSO, *T *is absolute temperature (K), *A *is a coefficient, *E *is the activation energy, and *R *is the universal gas constant (Table 1).

The viscosity of the OES at different values of χ _[AMIM]Cl _at the dissolution temperature (110°C) was calculated according to the Grunberg-Nissan mixing law [40]:

(6) $$\text{ln}\eta_{mix}=\overset{n}{\underset{i=1}{\sum }}x_{i}\;\text{ln}\;\eta_{i}$$

where *η_mix _*is viscosity of the OES; *x_i_*, and *η_i _*are mole fraction, and viscosity of component *i*.

### Number-average degree of polymerization (DP_n_) of cellulose

DP_n _of the cellulose sample was calculated as [32]:

(7) $$\text{D}{\text{P}}_{\text{n}}\text{ = }\frac{\text{glucosyl}\;\text{monomer}\;\text{concentration}}{\text{reducing - end}\;\text{concentration}}$$

The glucosyl monomer and reducing-end concentrations were measured by the phenol-sulfuric acid [41] and modified 2,2'-bicinchoninate methods [32], respectively. The samples were performed in triplicate.

### Measurement of specific surface area

The specific surface area of cellulose was determined by the BET method using gas adsorption (Tristar II 3020; Micromeritics Instrument Co. Ltd, Northcross, GA, USA). Samples were degassed at 100°C for 3 hours before analysis. Nitrogen with a relative pressure of 0.05 to 0.985 was used for the analyses.

### Enzymatic hydrolysis and glucose concentration

Enzymatic hydrolysis of cellulose was carried out with a substrate concentration of 0.4% (0.04 g) in a 50 mL Erlenmeyer flask containing 9.96 mL sodium citrate (50 mmol/L, pH 4.8) reaction buffer with *Trichoderma reesei *cellulase (2 mg powder per gram of cellulose; > 30 filter paper units (FPU)/mg powder; Bomei Biotech Co. Ltd, Heifei, Anhui, China), and *Aspergillus niger *cellobiase (Novozyme 188; Sigma-Aldrich, St Louis, MO, USA), a β-glucosidase enzyme (0.256 g solution per gram cellulose, giving an approximate β-glucosidase activity of 250 pNGU per gram solution. pNGU is defined as the number of μmol of *p*-nitrophenol produced per minute with *p*-nitrophenyl-β-D-glucopyranoside as substrate catalyzed by β-glucosidase at 50°C.).

Tetracycline 400 μg, and cycloheximide 300 μg were added to prevent bacterial growth during digestion. The sample in the flask was incubated at 50°C with shaking at 100 rpm for 3, 6, 12, 24, 48, and 72 hours. After enzymatic hydrolysis, a sample (150 μL) of the supernatant from the product mixture was transferred to a 1.5-mL Eppendorf centrifuge tube (Shanghai Sangon Biotech, China), and separated at 12,000 rpm (approx.13400 × g) for 10 minutes. The concentration of glucose in each sample was measured in triplicate using a biosensor analyzer with immobilized glucose-oxidase membranes (SBA-40D; Shandong Key Laboratory of Biosensor, Jinan, China). Each sample was diluted until its concentration was within the linear range of 0 to 100 mg/dL before analysis.

Untreated, hot-water-treated, and regenerated cellulose samples at a given χ _[AMIM]Cl _were prepared in duplicate. Three parallel runs for hydrolysis were conducted for each of the two prepared samples, separately. The reported hydrolysis yield was the average of the six results for the two prepared samples.

The hydrolysis (glucose) yield was calculated as follows:

(8) $$\text{Hydrolysis yield (\% ) = }\frac{\text{amount of glucose in the reaction system (g)}}{\text{amount of cellulose added (g)}}\times 0.9\times 100$$

Standard deviation (*SD*) was calculated as follows:

(9) $$SD\text{ = }\sqrt{\frac{\sum_{\text{i}}^{\text{n}}{\text{(}{\text{X}}_{\text{i}}\text{ - }{\text{X}}_{\text{m}}\text{)}}^{\text{2}}}{n}}$$

where X_m _is the root mean square of all X_i _values in the set, X_i _is a measured value from the set, and *n *is the number of samples in the set [42].

### Data treatment

Ohmine *et al. *proposed an empirical equation to describe the kinetics of enzymatic hydrolysis [34]:

(10) $$\text{X}=\frac{1}{k}\times \text{ln (1}+k\times v_{0}\times \tau \text{)}$$

where X is the hydrolysis yield (%), *k *is the rate retardation constant, which represents the change of hydrolysis rate, *v_0 _*is the initial hydrolysis rate (%/hour), and τ is the hydrolysis time (hours). In accordance with this equation, the experimental hydrolysis yields and times were used to determine *k*, and *v_0 _*via nonlinear curve fitting with Origin software.

Based on the score differences calculated by the Kruskal-Wallis test, we used the Student-Newman-Keuls (SNK) test to implement the multiple comparison to infer whether hydrolysis yield differed between cellulose samples, and found that *k*, and *v_0 _*differed between the different OES preparations at various χ _[AMIM]Cl _values from 0 to 1.0. Pearson's *r *was used to evaluate the canonical correlation analysis between χ _[AMIM]Cl _and the recovery rate of regenerated cellulose or speciﬁc surface area. The Kruskal-Wallis test was used to determine the significant differences of recovery rate, DP, and hydrolysis yield between the celluloses regenerated from the OES concentrations with various χ _[AMIM]Cl _values. All the analyses mentioned above were performed using SAS software (version 9.0; SAS Institute Inc., Cary, NC, USA). *P *< 0.05 was considered significant.

## List of abbreviations

[AMIM]Cl: 1-allyl-3-methylimidazolium chloride; BET: Bruner: Emmett: and Telle; CI: crystallinity index; DP: degree of polymerization; DP_n_: number-average DP; DMSO: dimethyl sulfoxide; IL: ionic liquid; OES: organic electrolyte solution; χ _[AMIM]Cl_: molar fraction of [AMIM]Cl per OES; XRD: x-ray diffraction.

## Competing interests

XFT and ZF hold a Chinese patent (application number 201110227943.4) related to pretreatment of crystalline cellulose by organic electrolyte solutions for enzymatic hydrolysis. ZF has developed a technique to rapidly dissolve and hydrolyze wood in hot water.

## Authors' contributions

XFT, and ZF (supervisor) conceived of the study. XFT carried out pretreatment tests, performed the statistical analyses, and drafted the manuscript. ZF participated in the test design and supervision, and helped to draft the manuscript. DJ carried out the hydrolysis experiments. XYS conducted the DP_n _analysis, and participated in the XRD and BET measurements. All authors read and approved the final manuscript.
